# Modeling the relationship between safety culture and safety performance in oil and gas using PLS-SEM

**DOI:** 10.3389/fpubh.2026.1811857

**Published:** 2026-06-05

**Authors:** Gehad Mohammed Ahmed Naji, Ahmad Shahrul Nizam Isha, Muhammad Shoaib Saleem, Yulita Hanum P Iskandar

**Affiliations:** 1 Graduate School of Business, Universiti Sains Malaysia, USM, Penang, Pulau Pinang, Malaysia; 2 Department of Management and Humanities, Universiti Teknologi PETRONAS, Seri Iskandar, Malaysia; 3 INTI International University Persiaran Perdana BBN, Nilai, Negeri Sembilan, Malaysia

**Keywords:** employment protection, oil and gas sector, safe working environment, safety culture, safety performance, SEM and PLS

## Abstract

**Introduction:**

This study examines the impact of safety culture on safety performance in Malaysia’s upstream oil and gas sector. It specifically investigates how key dimensions of safety culture—management commitment, leadership, employee involvement, work environment, and organisational communication—contribute to reducing workplace risks and improving safety outcomes.

**Methods:**

A quantitative, cross-sectional survey design was employed. Data were collected from 371 employees working in Malaysia’s upstream oil and gas industry. Structural Equation Modelling (SEM) was used to test the hypothesised relationships and evaluate the overall model fit.

**Results:**

The findings indicate that safety culture has a significant and positive effect on safety performance. All examined dimensions—management commitment, leadership, employee involvement, work environment, and organisational communication—were found to be strong predictors of improved safety outcomes. Organisations with a well-developed safety culture reported fewer incidents, more effective safety practices, and safer working conditions.

**Discussion:**

The results highlight the critical role of fostering a strong safety culture in high-risk industries. Practically, organisations can enhance safety performance by strengthening leadership involvement, improving communication, and encouraging employee participation in safety practices. However, the study is limited by its cross-sectional design, which restricts causal interpretation, and reliance on self-reported data within a specific industry context. Despite these limitations, the study contributes to the safety management literature by providing empirical evidence of the relationship between safety culture and safety performance in the Malaysian oil and gas sector, offering both theoretical insights and practical guidance for industry practitioners.

## 1 Introduction

The oil and gas sector is a complex and high-risk industry involving fossil fuel exploration, production, refining, and distribution. The nature of the industry, including the use of heavy equipment, hazardous chemicals, and extreme environmental conditions, means that safety is a critical aspect of operations (1). Despite technological advances and safety regulations, the oil and gas industry still experiences safety incidents, which can have severe consequences for workers, the environment, and the public. Research has shown that safety culture is crucial in improving safety performance in the oil and gas industry.

A positive safety culture is characterized by a solid commitment to safety at all levels of the organization, open communication channels, a willingness to learn from mistakes, and a focus on continuous improvement (2). In contrast, a negative safety culture is marked by complacency, a lack of safety leadership, and a failure to prioritize safety over other business objectives. Recent studies have highlighted the impact of safety culture on safety performance in the oil and gas industry. For example, a survey by Stemn et al. (3) examined the relationship between safety culture and safety performance in the offshore oil and gas sector. The study found that a positive safety culture was associated with fewer safety incidents and lower severity of incidents.

Safety performance is critical to organizational functioning, particularly in high-risk manufacturing, construction, and aviation industries. Several factors contribute to safety performance, including corporate culture, work environment, organizational communication, leadership, and employee involvement. This study empirically examines the relationship between management commitment, work environment, organizational communication, leadership, and employee involvement toward safety performance (4).

Management commitment is the degree to which management actively promotes and supports safety performance. Several studies have found a significant positive relationship between management commitment and safety performance. Found that management commitment was positively associated with employee safety behaviors and attitudes, and reduced rates of accidents and injuries. Probst and Brubaker (5) also found that management commitment was positively associated with safety performance in the US manufacturing industry. The work environment is another factor that affects safety performance. A positive work environment can lead to increased safety performance. A positive safety culture, which reflects the work environment, was associated with improved safety performance in the US construction industry.

Organizational communication is also a critical factor that affects safety performance. Effective communication within the organization can lead to increased safety performance. A study by Kalteh et al. (6) found that open and frequent communication between supervisors and employees was positively associated with safety performance in the Chinese manufacturing industry. Leadership is another factor that affects safety performance. Leaders play a vital role in promoting safety within an organization. A study by Grill et al. (7) found that safety leadership was positively associated with safety performance in the UK construction industry.

Employee involvement is also an essential factor that affects safety performance. Safety program employees are more likely to engage in safe work practices. A study by Bayram (8) found that employee involvement in safety programs was positively associated with safety performance in the Greek manufacturing industry.

The literature suggests that management commitment, work environment, organizational communication, leadership, and employee involvement are all critical factors that affect safety performance. A positive work environment, effective communication, and leadership can increase safety performance (9, 10). Management commitment and employee involvement in safety programs are also essential components of an overall safety program. Further research is needed to identify the most effective strategies for promoting management commitment, a positive work environment, effective communication, leadership, and employee involvement in safety programs (11–13).

Although the oil and gas industry, especially the upstream sector, has immensely exerted its efforts toward active safety measures, incidents and accidents continue to occur, hence putting the lives of the workforce at stake and causing threats to the environment and other stakeholders as well (14). One of the potential reasons for such safety slacks in this industry could be associated with the poor understanding of the complex construct of safety culture (a multifaceted higher-order construct) and the understanding of the safety performances at multiple levels in the form of leading indicators of safety (15). The subtle difference between organizational culture as a whole, leadership practices, and operational realities might influence safety-related outcomes in the workplace, yet the exact mechanism is insufficiently explored. Homogeneous to the general culture at work, safety culture in particular (as a higher-order construct) is considered a critical factor in improving safety by mitigating risks at work by exerting its influence on performance-related outcomes, i.e., efficiency and reduced incident rates, positive safety initiatives, yet the effect of safety leading indicators remains elusive. Therefore, a thorough understanding and empirical assessment of the safety culture’s latent constructs, such as management commitment, work environment, organizational culture, employee involvement, and leadership with safety leading indicators, necessitates this research inquiry to advance the understanding of safety culture with safety-related outcomes in the oil and gas sector. Henceforth, this research is essential for the oil and gas upstream industry to explore the dynamic link between sub-dimensions of safety culture and safety performance through leading indicators via advanced analytical techniques such as Partial Least Square (SEM).

Companies prioritizing safety culture are more likely to achieve better safety performance and mitigate the risks associated with their operations. A positive safety culture requires a commitment to safety at all levels of the organization, open communication channels, and a willingness to learn from mistakes. Therefore, this study will empirically assess the impact of safety culture’s latent factor on safety performance over two dimensions, i.e., safety leading indicators in the upstream oil and gas industry of Malaysia.

Although the relationship between safety culture and safety performance is widely acknowledged, empirical studies that examine this relationship using higher-order safety culture constructs and safety leading indicators within the Malaysian upstream oil and gas industry are scarce. This study fills that gap by integrating multi-dimensional safety culture factors into a higher-order framework, applying advanced PLS-SEM techniques, and simultaneously evaluating leading indicators to capture a more comprehensive and predictive model of safety performance in one of the most hazardous and understudied industrial contexts.

## 2 Literature review

### 2.1 Conceptualization of safety culture and its dimensions

Safety culture is a sub-organizational culture that places high importance on safety beliefs, values, attitudes, perceptions, competencies, and patterns of behavior that determine the commitment to, and the style and proficiency of, an organization’s health and safety management (16). Organizations with a positive safety culture are characterized by communications founded on mutual trust, shared perceptions of the importance of safety, and confidence in the efficacy of preventive measures. Fan et al. (17) defined safety culture as “the product of individual and group values, attitudes, perceptions, competencies and patterns of behavior that determine the commitment to, and the style and proficiency of, an organization’s health and safety management.”

According to Schein (18), organizational culture comprises shared values, assumptions, and norms that guide behavior in organizations. Safety culture, therefore, represents a domain-specific subset of organizational culture that focuses on safety-related beliefs and practices.

According to Carthey (19) ‘Safety culture refers to employees’ vision of safety conditions which affect safety outcomes. Consistent with prior literature, in this study, safety culture refers to the employees’ perception of safety conditions at the workplace, affecting organizational safety effectiveness (16, 20). Also, in this study, safety culture refers to employee involvement, perceived risk, and emergency response, which will be measured using the Safety Culture Scale. The reason for choosing such a scale is that it represents certain sub-dimensions of safety culture.

**Figure 1:**
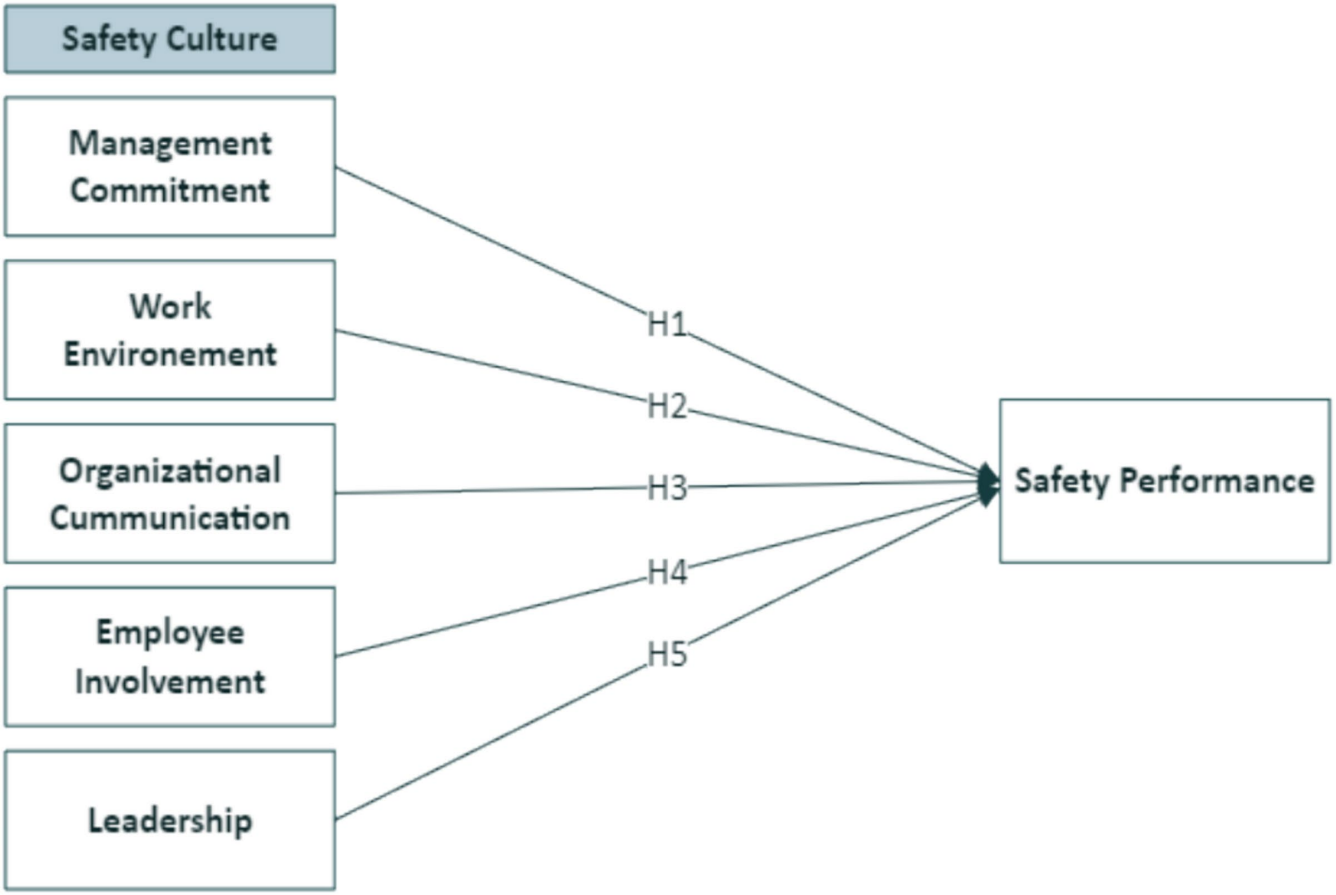
Research framework.

To guarantee that everyone understands the importance of safety and to alter attitudes and behaviors through the intrinsic and extrinsic components of corporate culture, everyone must participate. All organizational activities that involve the organization’s inner and external elements will be influenced by organizational culture (16, 21). As shown in Figure 1, the structural model illustrates the relationships between safety culture dimensions and safety performance, along with their corresponding path coefficients and statistical significance.

Due to this, employees in the organization will receive information in turn. Throughout the formation of a safety culture, both intrinsic and external cultural factors will impact the organization’s behavior. As a result, it increases overall acceptance of a safety culture. The safety system still applies to practice today, but it will work well after the organization establishes a safety culture. The cause may be viewed from several angles, but if the company cultivates a strong safety culture, the obstacle to adopting the safety system may be reduced (22, 23). Therefore, the safety culture dimensions will be described in detail in the following subsections.

For example, the health and safety culture comprises a range of materials that are obliquely referred to as indicators, principles, traits, characteristics, components, dimensions, and qualities. However, “dimension” was frequently used in academic writing to highlight the safety culture’s presumptive multidimensionality. Smaller sub-contents that make up a dimension are known as the attributes of that particular dimension. There is no consensus on the components that make up the safety culture construct, as seen in the conceptualizations and evaluation instruments that are now in use. According to Guldenmund (24), safety culture can include as few as two dimensions and as many as 19 dimensions, with little overlap in their names. Even when authors attempt to allude to the same safety culture contents, the labels used for these dimensions differ significantly from one author to the next. The widely acknowledged aspects of safety culture have been sought after in numerous reviews.

According to Sørensen (25), most studies agree that key aspects of safety culture include strong organizational communication, effective organizational learning, senior management’s commitment to safety, and a work environment that encourages identifying safety hazards. Furthermore, some assessments incorporate organizational and management factors, such as a leadership style that emphasizes participation. Wiegmann et al. (26), in another review, identified five components of safety culture: organizational commitment, management engagement, employee empowerment, reward systems, and reporting systems. The UK’s Health and Safety Executive, as cited by Edwards et al. (27) and Kalid et al. (28), highlighted five elements: safety leadership, two-way communication, employee engagement, an educational culture, and attitudes toward blame (also referred to as a just culture). Similarly, Choudhry et al. (29) described safety culture as comprising several elements: management concern for the workforce, commitment to safety, mutual trust and credibility between management and employees, workforce empowerment, continuous monitoring, corrective actions, system reviews, and ongoing improvements to enhance workplace safety.

#### 2.1.1 Management commitment and safety performance

Management’s commitment is crucial to establishing a solid safety culture within an organization, as emphasized by Brondino et al. (30). Encouraging management commitment and employee engagement in safety can improve an organization’s safety culture. This heightened security interest can result from increased awareness of employees’ roles in incident prevention and protection, as Feng et al. (154) and (172) highlighted. Management commitment has multiple aspects, including involvement from managers at all levels of the organization, active engagement in OHS-related activities, trust-building so that all employees view managers as dedicated to OHS, demonstration of OHS as a high priority, and serving as OHS role models. According to Van den Heuvel et al. (31), the relationship between safety culture and management commitment is powerful.

The concept of management commitment involves the active involvement of top-level management in critical areas such as safety, quality, environment, security, and organization programs, according to Johnson et al. (32). Additionally, management commitment is defined as “participating in and upholding behaviors that support others in achieving a goal.”

Generally, there are two ways to measure management commitment: by asking managers direct questions or by monitoring their commitment behaviors. In addition to direct managerial self-reports and behavioral observations, management commitment is frequently operationalized through employees’ perceptions, referred to as perceived management commitment. This approach reflects how workers interpret management’s priorities, actions, and responses toward safety issues, which ultimately shapes their safety attitudes and behaviors. Prior studies argue that employees’ perceptions of management commitment are particularly influential because they determine compliance, participation, and safety-related decision-making at the operational level (33, 34).

Perceived management commitment has been widely used in safety research, as workers are the primary recipients of management signals related to safety investments, communication, and enforcement. Empirical evidence demonstrates that perceived management commitment significantly influences psychological empowerment, safety behavior, and safety performance across high-risk industries (33–35).

Accordingly, the present study adopts the perceived management commitment approach, measuring employees’ evaluations of managerial actions and priorities toward safety. This approach is consistent with social exchange theory, which posits that employees reciprocate favorable organizational actions with positive safety behaviors when they perceive genuine management concern for their wellbeing.

According to Guo et al. (36), managers often do not admit their lack of safety commitment when asked, so behavior monitoring provides better proof of responsibility. Such commitment leads to higher motivation and concern for health and safety throughout the organization. The level of commitment is reflected in the proportion of resources (time, money, people) allocated to health and safety management and in the priority given to health and safety relative to production, cost, and other factors. Active involvement of senior management in health and safety is particularly critical. Previous studies have identified various factors contributing to safety culture, including individual attitudes toward safety and the physical work environment (34, 37).

According to Zohar and Luria (38), upper management’s safety policies can impact frontline supervisors’ actions at the work group level. When upper management prioritizes safety as a core value and becomes involved in daily safety-related activities and decision-making meetings, they establish guiding principles for frontline supervisors. This increased supervisory involvement can lead to greater employee awareness of safety risks in the workplace and greater compliance with safety procedures and regulations. As a result, frontline supervisors may take a more active role in supervising their employees by providing more task instructions and progress monitoring to meet the expectations and requirements set by upper management (39).

#### 2.1.2 Leadership and safety performance

The foundation of a safety culture begins with the leaders; they shape the culture that, in turn, affects employee behavior. Senior management can generally demonstrate their support for safety culture by providing necessary resources, personnel, and training for safety and conducting incident investigations (40, 41). As a company becomes more committed to safety, it may implement safety management systems, establish safety goals, and introduce additional safety protocols at the site level, including hazard analysis, behavior observation and feedback, incentives, action item tracking, and safety committees (42).

Organizational leaders must recognize the importance of improving safety regulations, as accidents can arise from a variety of causes. Research has shown that certain leadership practices contribute to enhancing workplace safety (41, 43). In healthcare settings, leadership is widely regarded as a key factor in driving improvements in quality and performance (44, 45). The role of leadership in fostering innovation has also gained significant attention, with studies emphasizing its influence, particularly in the oil and gas sector (46). Leadership not only shapes an organization’s capacity for innovation but also creates an environment conducive to innovation, especially by promoting organizational learning (47).

Leadership plays a pivotal role in improving workplace safety and addressing the impact of psychosocial hazards on employees’ mental health (48). Supportive, trustworthy leadership behaviors, along with providing constructive feedback, are linked to enhanced employee wellbeing and better stress management (49, 50). Over time, organizational values and practices have evolved from focusing solely on eliminating physical risks to addressing work demands that may lead to human errors. This is achieved by adopting proactive systems to enhance workplace conditions (49, 51). This shift embeds safety as a core organizational value and a fundamental aspect of operations. To foster such a culture, managers at all levels must demonstrate leadership in workplace safety by setting a strong example. While some individuals may naturally possess leadership qualities, others can become effective safety leaders. It is primarily behavior, rather than inherent personality traits, that defines an effective leader (52).

#### 2.1.3 Employee involvement and safety performance

Involvement is essential to the organization’s safety culture and is associated with several safety performances and outcomes (53). According to research, safety culture helps reduce risk by involving all organization members in its implementation (3, 54). However, a strong safety culture emphasizes being open to addressing issues and taking prompt action rather than necessarily leading to zero errors (55, 56). One of the factors cited by Oedewald and Reiman (57) as indicative of a strong safety culture is the management’s outward demonstration of its dedication to safety involvement. Managers are accountable for instilling strong safety awareness among their employees, instructing them on handling emergencies, and reducing risk in the workplace because they have been given a crucial role in creating a safety culture (58, 59). In any case, workers cannot fully protect themselves against the possibility of being engaged in an accident by awareness alone. Wu et al. (173) state that an organization’s commitment to the management and employees’ risk perceptions is crucial. Employees must be sensitive to risk perception and aware of ways to reduce it by instilling a standard value and confidence in safety.

Employee involvement is linked with safety leading indicators intricately within the organizational context. In order to form the bedrock of the leading indicators, the active participation of employees at all levels through safety engagement by developing and implementing proactive safety measures is inevitable. In order to produce a robust and sustained safety culture, the perspective of the frontline employees in the oil and gas industry in identifying potential challenges and hazards through self-driving initiatives and mitigation techniques is essential. Active involvement and contributing positively to safety-leading indicators, e.g., employees’ suggestions for safety, participation and involvement in training, and periodic cultural assessment of safety, may play critical roles in improving safety culture (60). Also, the enhanced safety awareness of an employee and compliance toward safety procedures could be associated with active involvement that may ultimately influence leading indicators, hence improving the company’s injury rate and overall safety performance.

The employees can grasp the high value of safety in their business due to their active involvement, strengthening perceptions of the safety atmosphere. When workers express concerns about safety culture, those concerns are swiftly addressed. Recommendations from employees are welcome to increase workplace safety. Even minor safety regulations are carefully monitored by leaders to prevent violations. Oil and gas personnel pay close attention to and adhere to safety procedures due to these measures. The notion that even minor safety failures are taken seriously matures due to such activities. Workers’ safety awareness and convictions increase due to the increased focus on safety. The self-motivation to engage in safety procedures based on favorable safety culture beliefs significantly reduces occupational work-related accidents because occupational accidents are often modest (61, 62).

Work involvement is defined as one of the strategy’s goals: to promote worker involvement and consultation in health and safety matters throughout unionized and non-unionized workplaces of all sizes. We have set this goal simply because the evidence suggests that involving workers positively affects health and safety performance (63). In addition, work involvement is “the combined involvement of management and health and safety representatives in inspections, investigations, and risk assessments (64, 65).

The demands of work and involvement of workers, coupled with limited management and work-life balance activities, have been shown to increase stress levels among employees (66, 67). As a result, this study aims to enhance the comprehension and creation of safety attitudes that would decrease workplace accidents (68). Stress can cause depression, anxiety, and anger, leading to significant expenses burdening the company (69, 70). The literature suggests that psychosocial risks and hazards have worsened and become more severe. The organization’s regulations and structure protect employees from such risks or, at the very least, reduce their impact (71). Consequently, the organization’s safety culture is expected to significantly reduce work involvement and mitigate the risk of these hazards.

#### 2.1.4 Organizational communication and safety performance

Communication refers to the process of exchanging information and the communication environment in an organization. Manager commitment is the perception of the support and dedication of leaders toward their employees. Active measurement provides insights into accidents that occur within an organization, including injury frequency, severity, and unsafe behavior. Passive measurement involves employees’ perceived risk, attitudes toward risk, suggestions for safety improvement, safety training courses, policy communication, and safety commitment. Safety performance measures the self-reported rate of accidents and occupational injuries. Anvari et al. (72) conducted a study on safety in various workplaces such as manufacturing, construction, service, and transportation industries. The research found that safety performance, employee safety control, and self-reported occupational injury are critical indicators of workplace safety (73). Additionally, Johari et al. (74) found that communication protocols are also a crucial aspect to consider when addressing psychosocial hazards in the workplace.

Effective communication between managers and employees regarding health and safety has been emphasized as a critical factor in the success of safety interventions. Strong evidence links communication quality to occupational accidents (75). It is vital for health and safety objectives to be integrated into organizations at the senior management level and to be included in all team meetings (76). Organizational communication regarding assessment and intervention is crucial to convey management’s commitment to addressing the issue, ensuring adequate worker participation, and enhancing employee performance. Effective communication is also essential to the logistics of implementing risk management processes.

With reference to safety indicators, passive measurement includes various aspects such as the perceived risk of employees, their attitudes toward risk, suggestions for safety improvement, safety training courses, policy communication, and safety committees (77). Huang et al. (78), the self-reported rate of accidents and occupational injuries is a way to gauge safety performance. Huang et al. conducted a study on safety across different industries, including manufacturing, construction, service, and transportation, and found that safety performance, worker safety control, and self-reports can impact the organizational culture and atmosphere (79, 80).

Further, Johari et al. (74) have studied communication rules concerning psychological risks. Effective communication between managers and employees regarding health and safety issues has been recognized as crucial for the success of safety interventions. Consistent evidence suggests a significant correlation between communication quality and occupational accidents. To ensure the success of safety measures, senior management must incorporate health and safety goals into organizational objectives, including team meeting agendas (76). The communication of safety evaluations and interventions throughout the organization is essential to demonstrate management’s dedication to addressing safety issues (81). Moreover, effective implementation of risk management processes and adequate worker participation must be ensured.

Effective communication channels facilitate the dissemination of safety-related information, policies, and procedures to employees, starting with leading indicators. Enhancing employees’ understanding of safety procedures can be achieved by ensuring employers emphasize transparent and clear communication about safety requirements, protocols, and potential hazards. As a result, proactive safety behaviors are promoted, creating a culture where employees are empowered to identify and address safety issues before they escalate into incidents. Key indicators of an organization’s dedication to safety communication and its impact on proactive safety measures include metrics such as the frequency and quality of safety communications, attendance rates at safety meetings, and employee evaluations regarding the clarity of safety instructions. These measurements consider how safety communication affects safety measures (82).

Internal communication in a business might impact safety performance metrics that are falling behind. Timely and efficient communication following events or near misses is crucial to promptly commence investigations, implement corrective measures, and prevent future recurrences. By creating clear communication channels, relevant information about incidents, lessons learned, and corrective actions is quickly shared with all levels of the business. This facilitates a comprehensive analysis of the contributing causes and enables the implementation of precise treatments to tackle fundamental safety issues. According to literature, the success of corporate communication in lowering risks and improving safety outcomes can also be assessed by the company’s performance on lagging indicators such as incident reaction times, the thoroughness of incident investigations, and the effectiveness of corrective actions (83).

#### 2.1.5 Work environment and safety performance

The work environment at a company is a crucial issue for management to consider, specifically in the oil and gas industry. The working environment directly affects the employees who carry out the production process, even though it does not carry out the manufacturing process. The setting in which employees carry out their daily tasks is referred to as the workplace. According to the Cheese Swiss Model, the working environment comprises design, protective gear, and guarding. As mentioned in the previous part, the causes of offshore accidents worldwide are weather and defective equipment (84). First, it is necessary to establish a workplace that supports everyone’s safety and wellness. Standardized processes for the upstream oil and gas sector are created to assist an organization’s capacity to function safely and successfully handle any contingencies (85). The preconditions of the working environment to achieve safety performance are organizational characteristics (such as leadership, supervision, training, and job design) and a safety culture (86). Therefore, the work environment has a strong relationship with safety culture in the success of occupational health and safety performance (87, 88).

According to Pawirosumarto et al. (89), the work environment is where employees perform their duties, and it can either positively or negatively affect their ability to meet their objectives. A favorable work environment can enhance employment continuity, whereas an unfavorable work environment can hinder job continuity. Additionally, a work environment is where employees work together to accomplish organizational objectives (90, 91).

The work environment encompasses the physical location and surroundings where individuals perform tasks and interact with others. The quality of the work environment has a significant impact on employees’ overall productivity. The work environment includes a range of factors that positively or negatively affect individual performance, such as procedures, systems, structures, tools, or locations in the workplace (92). Workplace policies, culture, working relationships, resources, and internal and external environmental elements also impact employees’ roles (93). Processes, procedures, structures, tools, or situations in the workplace that affect individual performance positively or negatively are part of the work environment, which heavily influences employee performance and productivity (94, 95).

The quality of one’s life is affected by one’s work situation, and joblessness is associated with a greater likelihood of experiencing common mental disorders (96). However, a hostile psychosocial work environment can harm workers’ mental health (67). The workers’ cognitive functions are under increasing pressure due to the need to work faster, the proliferation of highly skilled jobs, and the growing use of communication and information technology (97, 98).

Firstly, a positive work atmosphere might promote proactive protective measures, which can be considered as leading signs. Employees who prioritize safety activities are usually found in firms that prioritize creating a safe and supportive work environment. Key engagement indicators include active participation in safety training and safety committees, and a higher frequency of safety proposals or near-miss reporting. Fostering trust, promoting open communication, and empowering employees to take responsibility for safety are critical elements of a positive work environment that lead to a proactive safety culture.

The working climate is a factor that can impact the leading indicators of safety performance levels. Supportive environments are associated with lower stress levels, fatigue, and job dissatisfaction. These elements can contribute to a decrease in accidents and incidents. A supportive work environment can also positively influence lagging indicators, including injury rates, absenteeism due to illness or injury, and turnover rates, which are different yet close element of safety compared to leading indicators. A pleasant work environment promotes adherence to safety standards and timely reporting of occurrences by employees. This facilitates thorough examinations of incidents and the implementation of effective corrective measures to prevent future occurrences (99).

#### 2.1.6 Safety performance

Safety performance has been a significant focus of research in recent years due to its importance in ensuring the wellbeing of employees and reducing the cost of accidents and incidents in the workplace. Safety performance is an organization’s ability to manage safety effectively and efficiently (100). This literature review aims to provide an overview of recent research on safety performance.

Several factors have been found to impact safety performance, including safety climate, safety culture, safety leadership, safety training, and safety motivation. For instance, Arzahan et al. (101) found that safety culture significantly affects safety performance, and organizations with a favorable safety climate tend to have better safety performance. Another study by Hu and Morrow (102, 103) identified safety culture as an essential factor affecting safety performance, with a positive safety culture leading to better safety performance. Additionally, safety leadership has been found to influence safety performance, with good safety leadership leading to better safety performance (104).

Several methods have been used to measure safety performance, including leading indicators. In the past literature, lagging indicators, such as accident and injury rates, are reactive and indicate past safety performance. In contrast, proactive indicators, such as safety audits and training, can help organizations identify potential safety hazards before incidents occur (6). Furthermore, researchers have suggested using safety maturity models to measure safety performance. These models provide a comprehensive framework for assessing an organization’s safety performance based on several factors.

Several interventions have been suggested to improve safety performance, including safety training, safety culture improvement programs, and safety leadership development. A study by Namian et al. (105) found that safety training significantly improved safety performance in the workplace. Another survey by Cooper (106) suggested using safety culture improvement programs to improve safety performance. Safety leadership development has also been found to enhance safety performance, with leaders playing a crucial role in promoting a positive safety culture and motivating employees to comply with safety procedures (35).

In conclusion, safety performance is a crucial aspect of organizational performance, and several factors impact it. Safety climate, culture, leadership, training, and motivation affect safety performance. Furthermore, leading indicators and safety maturity models can help organizations measure their safety performance. Finally, interventions such as safety training, safety culture improvement programs, and safety leadership development can enhance safety performance in the workplace. In addition, the research hypotheses are listed in Table 1.

**Table 1:** Study research hypotheses.

No	Hypothesis
H1	“There is a significant relationship between management commitment and safety performance.”
H2	“There is a significant relationship between work environment and safety performance.”
H3	“There is a significant relationship between organizational communication and safety performance.”
H4	“There is a significant relationship between employee involvement and safety performance.”
H5	“There is a significant relationship between leadership and safety performance.”

From the theoretical underpinning view, this study uses social exchange theory (107) as an underpinning framework for the proposed model. Out of many theories, Social Exchange Theory (SET) was chosen to explain the research framework due to its intervention at the micro-level in the organization. On the contrary, organizational culture theory highlights collective elements such as shared values, beliefs, and group norms that affect overall and individual behaviors (18), a macro-level approach. On the other hand, social exchange theory (SET) talks about the reciprocal relationship between management and their employees and how management’s efforts through its managers enhance trust among employees, ultimately fostering cooperation and exchange. The interpersonal exchange between m and the workforce is highlighted through Social Exchange Theory (SET), which differentiates this from general organizational cultural theory.

In addition, the Theory of Planned Behavior (108), provides further support for the proposed relationships. According to TPB, employees’ safety behaviors are driven by their attitudes toward safety, perceived social expectations (subjective norms), and perceived behavioral control. Leadership communication, safety climate, and employee involvement directly influence these three components, shaping employees’ intention to behave safely and ultimately their actual safety behavior. Thus, TPB strengthens the theoretical justification for hypotheses related to employee involvement, leadership influence, and safety behavior outcomes.

Social Learning Theory (109), suggests that employees learn safety behaviors by observing leaders, supervisors, and coworkers. Leaders act as role models whose actions, communication patterns, and reactions to safety issues shape worker beliefs and behaviors. This positions leadership, communication, and organizational practices as behavioral models that influence safety outcomes, offering theoretical justification for hypotheses involving leadership and organizational communication.

Drawing from the Resource-Based View (110), safety culture represents a valuable, rare, and inimitable organizational resource that contributes to sustained performance advantages. A strong safety culture reduces operational disruptions, prevents incidents, improves employee wellbeing, and enhances organizational reliability. Consequently, RBV reinforces the conceptualization of safety culture as a strategic capability that directly enhances safety performance.

Organizational and individual wellbeing and safety are expected to be influenced by the underpinning role of social exchange theory for the five study dimensions of safety culture. Fostering a work environment filled with warmth and mutual trust/benefit amongst the workforce is pivotal to promoting work involvement and allocating power and resources to the workforce to participate in safety-related decision-making and initiatives practically. Further, the role of leadership in the purview of social exchange theory seems pertinent since their encouragement, communication, and efforts to establish a positive work climate also count for positive social interaction among employees. Based on the premise of social exchange theory, the role of leader in determining safety standards through their observance may also lead to a positive safety culture. The leader’s commitment is henceforth exerted through resource allocation, determining vivid safety goals and continuous evaluation of safety goals. In doing so, a leader can instill trust and honesty regarding safety at work through the organization, ultimately helping the workforce to exhibit their concerns and input to strengthen the positive safety culture.

Based on the theoretical propositions of social exchange theory (SET), reciprocity prevails regarding management actions if they exhibit positive workplace interventions, against which employees will display positive behaviors (107). For instance, specifically for the safety culture, the efforts and commitment shown by the management will foster a two-way situation, fostering a sense of obligation for the workforce toward safety behaviors. In terms of investments in safety equipment, gadgets, and training, organizational efforts toward safety will propagate the positive perception of management’s action, giving clues and signs to the employees that their organization is concerned about their safety and overall wellbeing (111). The investment and efforts of the management perceived by the workforce force them to participate in organizational safety by following safety directions, using protective gear, speaking about hazards and participating in the overall safety aspects of the organization (112). The role of leaders, especially transformational leaders, is crucial to fostering an environment where trust between management and the workforce is pivotal in driving employees toward safe behaviors. Observed behaviors of leaders, such as seeking advice from the workforce to enhance safety measures and actively listening and responding to them, will foster a positive culture, which will produce a safer environment (113). Literature supports this notion that when the workforce is psychologically and psychologically secure enough to express themselves, they are more inclined to reciprocate positively, which will be in harmony with management efforts for safety at work (114).

## 3 Materials and methods

Research methodology describes the steps followed to answer the research questions and objectives and explains the qualitative or quantitative research design followed in the study (171). The methodology selected plays a critical part in determining the results. The current study followed the positivist research paradigm, focusing on “quantification,” analysis of data collected, and theory testing (115). Statistical and analytical procedures assessed the relationships between variables (116, 117). In quantitative research design, two methodologies are usually conducted in succession: survey research and experimental research (118).

The current research followed a quantitative research design and employed questionnaire surveys for data collection because they provided “standardized information” to explain or study the relationship among variables of interest (119), making it the most appropriate current research method. Moreover, this study aimed to examine the effect of safety culture on safety performance via psychosocial hazards. Previous safety culture studies have primarily focused on quantitative methods and self-administered questionnaire techniques for data collection (120) because theories can only be verified with suitable quantitative methods (121). Therefore, the current study used a survey method to collect respondents’ data. The collected data were used to examine the impacts of safety culture on safety performance via psychosocial hazards.

### 3.1 Procedures

From Malaysia’s upstream oil and gas sector, 550 employees were selected for stratified sampling. Of the 500 questionnaires distributed, 423 were returned, but 23 were excluded due to incomplete data. After cleaning the data and removing any outliers, 371 questionnaires were considered for data analysis. The sample size is deemed adequate, with a 95% confidence interval and a 4% margin of error (122). The study involving human subjects was exempt from ethical review and approval since it complied with the local legislation and institutional criteria. The participants granted their written informed consent to partake in this study. Also, no minors were included as respondents to this study survey. As a result, the demographic details of the participants are presented in Table 2.

**Table 2:** Demographic information.

Demographic categories	Categories	Frequencies (*n* = 371)	Percentages %
Gender	Male	366	98.65%
	Female	5	1.35%
Age	20–29 Years	48	12.94%
	30–39 Years	159	42.86%
	40–49 Years	93	25.07%
	50–59 Years	57	15.36%
	60 years and above	14	3.77%
Marital status	Single	57	15.36%
	Married	288	77.63%
	Divorced	26	7.01%
Education	Graduate/Postgraduate	15	4.04%
	College	54	14.56%
	Secondary	290	78.17%
	Primary	12	3.23%

Stratified sampling was applied by dividing the population into four strata based on job categories (operators, technicians, engineers, and HSE professionals). These strata were chosen because risk exposure and safety responsibilities differ significantly across roles, and proportional samples were drawn from each group to improve representativeness.

### 3.2 Sample size

To determine the appropriate sample size for the study, G*Power version 3.9.2 was utilized for investigation (123). In social and behavioral research, choosing the sample size using the highest number of predictive correlations is a dependable and practical approach. This method requires a minimum power of 0.80 for sample size calculation (123). The study’s sample size was calculated using a capacity of 0.95 and the model’s highest number of predictors, which was three, instead of the standard minimum power of 0.80. With a medium effect size and a 0.05 probability of error, the minimum required sample size for the study was 119. However, the final sample size for the study was 371 individuals. Table 3. outlines the structure of the instruments used in this study. The questionnaire was designed based on an extensive review of the literature, resulting in the development of a comprehensive cross-sectional survey. Following a pilot study, necessary adjustments were made to refine the instrument. The main survey aimed to explore the influence of leadership, organizational communication, and work environment on psychosocial hazards. It was distributed to a large sample of employees in Malaysia’s upstream oil and gas sector. The sample size was determined using the Morgan table methodology as noted by Kyriazos (124), a Structural Equation Modeling (SEM) analysis requires a sample size exceeding 100 participants, (125) recommend at least 200 for a robust path model. A total of 371 valid responses were collected from employees in the upstream oil and gas industry. The questionnaire consisted of two sections: the first gathered demographic information about the respondents, while the second assessed the study variables using a five-point Likert scale, with response options ranging from “(1 = Never, 2 = Seldom, 3 = Neutral, 4 = Often, 5 = Always)”.

**Table 3:** Instrument’s structure.

Variables	Dimensions	No. of items	References
Safety culture	Management-Commitment (MC) Work-Environment (WE) Organizational-Communication (OC) Leadership (LS) Employee Involvement (EI)	32	(167, 168)
Safety performance (SP)		9	(169, 170)

This study explores the relationship between safety culture and safety performance within Malaysia’s oil and gas industry. By analyzing the framework’s components in the Malaysian context, the research aims to identify alternative approaches to enhancing safety, minimizing risks, and mitigating incidents.

The management commitment construct was measured using employees’ perceptions of managerial safety practices, consistent with prior studies on perceived management commitment in safety research. For this research, a “stratified random sampling technique” was utilized to select the sample from the population under study. Stratified random sampling involves dividing the population into distinct strata or groups and randomly assigning participants from each stratum (126). Four hundred twenty-three questionnaires were handed out to individuals working in Malaysia’s petrochemical oil and gas sector. After eliminating invalid surveys, 371 authentic questionnaires remained, indicating an 86.89% response rate. The study’s minimum sample size was calculated using “G* power software v3.1 University of Dusseldorf, Dusseldorf, Germany,” according to Maccallum et al. (127, 128).

## 4 Results and analysis

**Figure 2:**
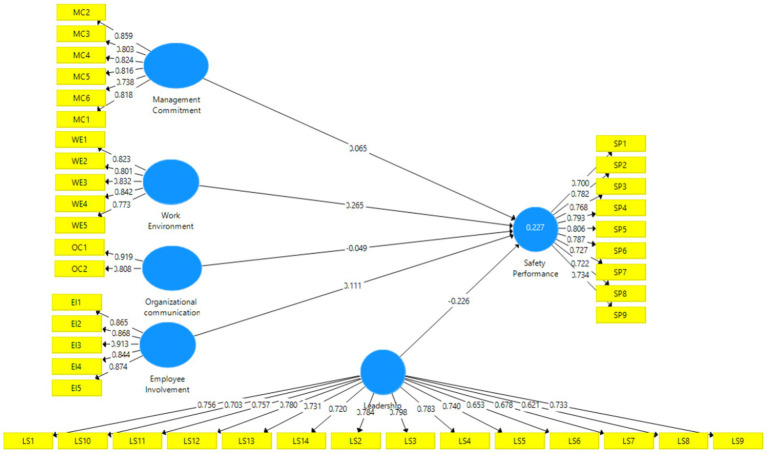
Structural model outcomes.

**Figure 3:**
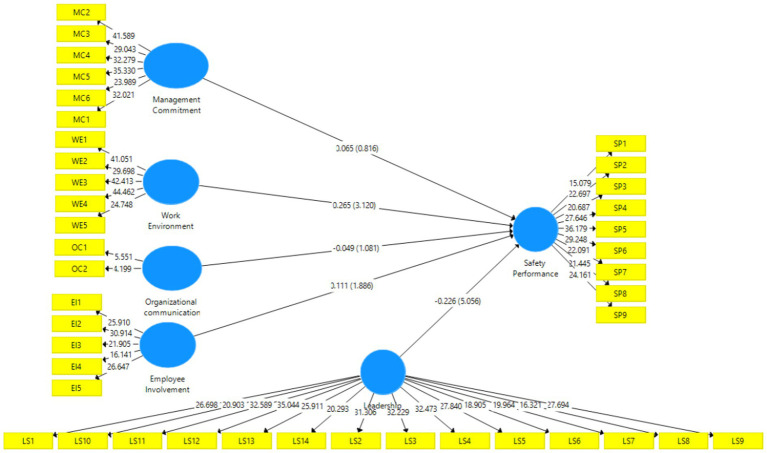
Bootstrapping analysis and *T*-values.

To assess the proposed model, the “Partial Least Square technique (Smart-PLS 3.2.7) software” was employed (129). We adopted the recommended two-step analytical procedure to evaluate both the structural and measurement models, as shown in Figures 2, 3 (130). We utilized “G* Power version 3.1.9.2 Faul et al. (123) to determine the appropriate sample size. According to the standard behavioral and social sciences guideline, a required sample size of 68 was selected for the study. The study included a total sample size of 371 employees, surpassing the minimum sample size requirement. However, this sample size was more significant than the optimal sample size of 100 for conducting (PLS-SEM) analyzes (131–133).

### 4.1 Convergent validity

The initial assessment focused on evaluating the measurement model by examining outer loadings (OL), Cronbach’s alpha (CA), construct reliability (CR), average variance extracted (AVE), and validity. The variance inflation factor (VIF) values for all constructs are presented in Table 3. To evaluate the internal consistency of the indicators, their values should meet or exceed the respective threshold values. An indicator such as Composite Reliability (CR) measures internal consistency and should surpass the minimum threshold value of 0.70 (134). The internal consistency of a construct is evaluated using composite reliabilities. The appropriateness of a construct is shown by an average variance extracted (AVE) value greater than 0.05 (135, 136). Newly developed items must have a factor loading greater than 0.5 for each item, while established items should have a factor loading of 0.6 or higher (137). Falk and Miller (138) suggest that any item with a factor loading below 0.6 should have an R2 of at least 0.1. Thus, the reliability of the contextual measures is not affected by the correlation coefficients of all constructs being more than 0.50, and the hypothesis remains valid. Additionally, Table 4, illustrates that the AVE values for all constructs are more significant than 0.5, indicating adequate convergent validity.

**Table 4:** The outcomes of PLS analysis on convergent validity and reliability.

Construct’s statement	Path relationship	SFL	CA	CR	AVE
	Management commitment		0.896	0.920	0.657
“In my workplace, the management acts quickly to correct safety problems”.	Mc1 ← MC	0.818			
“Management acts decisively when a safety concern is raised”.	Mc2 ← MC	0.859			
“In my workplace, management turns a blind eye to safety issues”.	Mc3 ← MC	0.803			
“Corrective action is always taken when I tell management about unsafe practices”.	Mc4 ← MC	0.824			
“In my workplace, managers/supervisors show interest in my safety.”	Mc5 ← MC	0.816			
“Management acts only after accidents have occurred.”	Mc6 ← MC	0.738			
	Work environment		0.873	0.908	0.663
“Operational targets often conflict with safety measures”.	We1 ← WE	0.823			
“Sometimes I am not given enough time to get the job done safely.”	We2 ← WE	0.801			
“Sometimes conditions here hinder my ability to work safely.”	We3 ← WE	0.832			
“I cannot always get the equipment I need to do the job safely.”	We4 ← WE	0.842			
“I feel safer in this place to work.”	We5 ← WE	0.773			
	Organizational communication		0.877	0.856	0.749
“There is good communication here about safety issues which affects me”.	Oc1 ← OC	0.919			
“Safety information is always brought to my attention by my line manager/supervisor.”	Oc2 ← OC	0.808			
	Leadership		0.933	0.942	0.537
“My senior managers/ leaders have established a safety responsibility system”.	Ls1 ← LS	0.756			
“My senior managers/ leaders express an interest in acting on safety policies”.	Ls2 ← LS	0.784			
“My senior managers/ leaders are concerned about safety improvement.”	Ls3 ← LS	0.798			
“My senior managers/leaders establish clear safety goals.”	Ls4 ← LS	0.783			
“My senior managers/ leaders coordinate with other departments to solve safety issues.”	Ls5 ← LS	0.740			
“My senior managers/ leaders explain the safety mission clearly.”	Ls6 ← LS	0.653			
“My senior managers/ leaders encourage workers to provide safety suggestions.”	Ls7 ← LS	0.678			
“My senior managers/leaders emphasize worksite safety.”	Ls8 ← LS	0.621			
“My senior managers/ leaders stress the importance of wearing personal protective equipment.”	Ls9 ← LS	0.733			
“My senior managers/ leaders encourage workers’ participation in safety decision-making.”	Ls10 ← LS	0.703			
“My senior managers/ leaders encourage workers to report potential incidents without punishment.”	Ls11 ← LS	0.757			
“My senior managers/ leaders show consideration for workers.”	Ls12 ← LS	0.780			
“My senior managers/ leaders trust workers.”	Ls13 ← LS	0.731			
“My senior managers/leaders praise workers’ safety behavior.”	Ls14 ← LS	0.720			
	Employee involvement		0.923	0.941	0.762
“Management always welcomes opinions from employees before making final decisions on safety-related matters.”	Ei1 ← EI	0.865			
“My company has safety committees consisting of representatives of management and employees.”	Ei2 ← EI	0.868			
“Management promotes employees’ involvement in safety-related matters.”	Ei3 ← EI	0.913			
“Management consults with employees regularly about workplace health and safety issues.”	Ei4 ← EI	0.844			
“I am involved in informing management of important safety issues.”	Ei5 ← EI	0.874			
	Safety performance		0.908	0.924	0.575
“Formal occupational health and safety audits at regular intervals are a normal part of our workplace.”	Sp1 ← SP	0.700			
“Everyone at this workplace appreciates ongoing occupational health and safety improvement in this workplace.”	Sp2 ← SP	0.782			
“Health and safety are important as product quality in the way the work is done.”	Sp3 ← SP	0.768			
“Workers and supervisors have the information they need to work safely.”	Sp4 ← SP	0.793			
“Employees are always involved in decisions affecting their health and safety.”	Sp5 ← SP	0.806			
“Those in charge of occupational health and safety have the authority to make the changes they have identified as necessary.”	Sp6 ← SP	0.787			
“Those who act safely receive positive recognition.”	Sp7 ← SP	0.727			
“Operating procedures are followed during the start-up operations of the unit.”	Sp8 ← SP	0.722			
“Procedures are followed during the emergency shutdown of the unit.”	Sp9 ← SP	0.734			

SIL, Standardized Indicator Loadings; CA, Cronbach’s Alpha; CR, Composite Reliability; AVE, Average Variance Extracted; LEP, Level Explanatory Power.

### 4.2 Discriminant validity

As shown in Table 5, the SIL values ranged from 0.621 to 0.919, all above the threshold of 0.650. The Cronbach’s alpha (CA) values ranged from 0.873 to 0.933, exceeding the 0.700 benchmark. Construct reliability (CR) values were between 0.856 and 0.941, surpassing the threshold of 0.650, while the Average Variance Extracted (AVE) values ranged from 0.537 to 0.749, all above the recommended minimum of 0.50 (139). These values were consistently higher than the correlations between other variables, confirming the model’s discriminant validity.

**Table 5:** The discriminant validity results.

Construct’s statement	1	2	3	4	5	6
1. Employee involvement	0.873					
2. Leadership	−0.143	0.733				
3. Management commitment	0.212	−0.251	0.811			
4. Organizational communication	0.004	0.16	−0.049	0.866		
5. Safety performance	0.218	−0.32	0.349	−0.101	0.758	
6. Work environment	0.231	−0.203	0.762	−0.047	0.388	0.814

The measurement model assesses two types of validity: convergent validity (CV) and discriminant validity (DV). Convergent validity refers to how closely related items align with the construct they measure (140). As indicated in Table 5, discriminant validity describes the degree to which constructs are distinct from each other. This was evaluated using the Fornell and Larcker (141) criterion, which specifies that the square root of each construct’s AVE should be greater than its correlations with other constructs.

### 4.3 Hypothesis outcomes

We anticipated a significant relationship between safety culture factors and safety performance. This was confirmed by the results for Hypothesis 1, which indicated a positive link between management commitment (MC) and safety performance (SP) (*β* = 0.065, *p* < 0.05). Hypothesis 2 predicted a positive correlation with the work environment, and this was also supported by the results (*β* = 0.265, *p* < 0.05). Hypothesis 3, however, proposed a non-significant relationship between organizational communication and safety performance. The results showed a negative coefficient (*β* = −0.049, *p* > 0.05), which was contrary to expectations and previous literature, as shown in Table 6. Therefore, we reject this hypothesis because it might not be enough to reduce near-miss incidents. Hypothesis 4 predicted a significant relationship, but not between employee involvement and safety performance (*β* = 0.111, *p* > 0.05). Therefore, we reject this hypothesis since it was out of expectation. Hypothesis 5 predicted a non-significant relationship between leadership and safety performance (*β* = −0.226, *p* < 0.05), supported by our expectation. We used the approach Preacher and Hayes (142) used to conduct the analysis.

**Table 6:** Findings of hypothesis testing.

Hypothesis	H1	H2	H3	H4	H5
Path relationship	MC → SP	WE → SP	OC → SP	EI → SP	LS → SP
Path coefficients β	0.065	0.265	−0.049	0.111	−0.226
STDEV	0.079	0.085	0.046	0.059	0.045
*T* values	0.816	3.120	1.081	1.886	5.056
*p* values	0.001	0.002	0.280	0.060	0.000
Significance level	***	***	*	*	***
Results	Supported	Supported	Not supported	Not supported	Supported

## 5 Discussions and conclusion

The main objective of this study was to assess the impact of safety culture dimensions on workplace safety performance. Following the Social Exchange Theory (SET) (143), the study explored the link between safety culture dimensions and safety performance by formulating a specific hypothesis connecting management commitment, work environment, organizational communication, leadership, and employee involvement to safety performance. The suggestion was made that by having a more robust safety culture, more resources would be available to improve safety performance by reducing the number of accidents and fatalities among employees. As accidents are facilitated through a safety culture, workers can allocate their saved resources toward obtaining and improving safety performance. Our study makes a significant contribution to the field of theory in two ways. Firstly, it enhances the safety culture theory in occupational safety literature (144, 145), and secondly, it emphasizes personal safety by stressing organizational efforts through its positive safety culture for reducing workplace accidents and deaths (146, 147).

Additionally, the results indicate that to promote employee safety performance (in terms of safety leading indicators), improving the general safety culture is more important than solely concentrating on physical safety (147). There is a recommendation that upcoming research should integrate measures of safety climates to enhance our comprehension of their influence on workplace safety performance (148, 149). Our study’s findings indicate that future research could enhance its comprehension of the functions and guidelines of workplace safety by incorporating safety climate and safety performance metrics. Our investigation revealed that both safety culture and safety performance play a significant role in reducing accidents and fatalities, aligning with prior research (150–153). Our study makes a valuable contribution to the safety culture literature by demonstrating that a psychologically supportive culture can mitigate psychological distress and prevent fatalities, even in high-risk work settings.

Exploring the connection in high-risk scenarios such as accidents and fatalities has been a relatively uncharted area in research (154–157). Moreover, most previous studies on workplace safety have primarily employed injury as a metric, with a limited exploration into how safety culture dimensions influence worker safety performance. Our study addresses a significant void within occupational safety and health (3, 158). “Our findings, grounded in the safety performance concept, reveal that enhancing safety communication leads to enhancements in employee safety performance and safety culture. While our assessment centered on safety leadership, it underscored the importance of tailoring safety approaches to the specific contextual demands of different businesses concerning workplace safety”.

Regarding the findings of our study on leadership and its impact on safety behaviors, it was unexpected to find a negative relationship. One of the possible reasons for this could be the leadership style in the organization and its relevance to the culture. In a competitive oil and gas environment, it may be the manager’s discretion to choose between a transactional or authoritarian leadership style/approach based on short-term results and productivity, thereby suppressing the workforce’s priority for safety over productivity. Such a situation may foster a culture where pressure is exerted toward productivity to meet targets, compromising safety and initiating safety behavior. One of the other reasons could be the inconsistent communication, messages and cues from management concerning the priority of safety over productivity. In case of a leader’s communication failure to express his support for safety initiatives, the inclination of the workforce and its priority toward safety may be compromised.

Our findings suggest that in high-risk industries like oil and gas, addressing psychological factors is as essential as managing physical conditions to improve worker safety performance. This research contributes significantly to the field of occupational safety by highlighting the importance of a holistic approach. In industries with challenging work environments, such as oil and gas, companies should emphasize safety culture elements to strengthen employee safety outcomes. Future studies on safety enhancement strategies should include both psychosocial and physical safety aspects.

### 5.1 Theoretical implications

The safety culture of a company influences the safety performance of its employees. Based on the “social exchange theory,” this study confirms that safety culture significantly affects safety performance. This research contributes to the existing knowledge by examining the direct relationship between safety culture dimensions and safety performance and the indirect impact of safety culture on safety performance. In addition, there is a lack of research on safety culture in Asian settings, particularly in Southeast Asian countries such as Malaysia. Therefore, this research provides valuable insights for practitioners and academics regarding safety interventions and future studies. Our study identifies and investigates a sizable research gap around safety culture in Asian environments, particularly in the context of Southeast Asian nations like Malaysia. The lack of previous studies in this particular geographic and cultural context highlights the uniqueness and significance of our work.

The study offers valuable insights from the perspective of organizational behaviors and safety management through social exchange theory. The study underscores the importance of workforce involvement throughout safety practices to strengthen the urgency and need for individual ownership and responsibility, utilizing social exchange principles. Also, the reciprocal role of leadership in enhancing safety culture and explicitly helping the workforce to highlight leading indicators for safety helps prioritize safety competencies and individual behaviors. Exploring the association between management commitment and its effect on safety performance through leading safety indicators also underscores the integration of safety goals and strategic alignment, which helps enhance prevailing safety norms and culture. Since learning also comes from the social exchange between the workforce, the role of organizational communication in straightforward communication channels may also facilitate the dissemination of safety-related information, consequently fostering an open culture for dialogue and improvement. Moreover, social exchange theory posits reciprocity between individuals and their surroundings; thereby, the work environment may also underscore creating a flexible, supportive and safe environment for all to initiate any idea and change, ultimately benefiting overall safety performance. Study findings support the theoretical proposition of the social exchange theory and support the notion that exchange is expected to occur from both ends, the worker and the employer (organization), and that exchange can be used to enhance organizational and individual safety performance.

Consequently, this research opens new scholarly avenues and provides practitioners with priceless insights. These observations also apply to safety interventions; they offer practical methods for fostering a safety culture and improving safety performance. Furthermore, the study establishes a framework for further research, promoting an ongoing investigation of the dynamics of safety culture in various organizational and cultural contexts.

### 5.2 Practical implications

The findings of this study have important implications for industry professionals. While a positive safety culture has traditionally been viewed as essential for maintaining safe working conditions (159, 160) our results suggest that achieving workplace safety requires attention to both physical safety measures and safety culture. Managers should foster a strong safety culture to enhance employee safety performance and support their psychosocial wellbeing. By cultivating an environment where employees feel that management values their mental health, minimizes excessive workloads, and maintains a psychologically safe workplace, organizations can reduce incidents, fatalities, and stress levels. Such a work environment also enables employees to retain safety information more effectively, engage in safety practices, and implement them, ultimately leading to increased productivity.

According to Cooper (161), the literature on safety culture suggests practical steps organizations should implement to build a strong safety culture. Enforcing safety culture standards through legislation, especially in high-risk sectors like oil and gas, can be an effective method for ensuring compliance (162). Other effective strategies include integrating safety performance into evaluations and promoting individuals who demonstrate strong adherence to safety culture policies to senior positions (163). Our findings further indicate that high-risk industries, such as oil and gas, should prioritize worker mental health and safety even above production goals to improve safety outcomes. Given the positive relationship between safety culture dimensions and safety performance, companies in this industry require additional insights on how to leverage these dimensions to enhance the reporting of leading indicators and to support workforce cooperation on addressing lagging indicators. It is also important to note that measures that require leadership to actively encourage reporting and collaborate on addressing lagging indicators are essential for fostering a robust safety culture.

### 5.3 Study limitations and future direction

We must exercise caution when interpreting our findings, as our study has a cross-sectional design. We opted for a cross-sectional approach for two main reasons. Firstly, gathering data from Malaysia’s petrochemical oil and gas industry posed significant challenges due to the time and effort required to access local businesses. Secondly, researchers encountered difficulties in data collection multiple times due to a lack of support for research efforts in Malaysia (164). While cross-sectional research can be valuable in the preliminary phases of a study (165), a longitudinal design becomes essential for establishing causal relationships and mediation. In future studies, it would be advisable to incorporate measures of various cultural factors, particularly safety culture, to better understand their individual and comparative impacts on workplace injuries.

Likewise, a study examining psychological wellbeing was conducted (166). Given that our analysis exclusively drew data from Malaysia’s oil and gas industry, it is essential to exercise caution when generalizing these findings to other settings. It would be intriguing to explore whether individuals in diverse industries and professions with varying job demands experience similar effects related to unsafe acts and conditions (UAUS). Our research did not directly assess employee resources; however, we explored a theoretical framework grounded in social exchange theory (SET). We propose that establishing a comprehensive metric for personal resources within a psychosocially and socially secure culture could significantly contribute to the safety literature and deepen our understanding of SET. Conducting a comparative analysis of safety performance in safe versus unsafe workplace cultures could further refine safety theory while providing practical guidance for organizations and managers.

## Data Availability

The raw data supporting the conclusions of this article will be made available by the authors without undue reservation.
